# The Combination of Tin and Gold as a Filling Material

**Published:** 1891-02

**Authors:** H. F. Hamilton


ARTICLE IX.

### THE COMBINATION OF TIN AND GOLD AS A FILLING-MATERIAL.

BY DR. H. F. HAMILTON.

**[Read at a Meeting of the American Academy of Dental Science, May 7, 1890.]**

When Dr. Charles H. Abbott, son of the late Dr. Frank Abbott, of Berlin, was in this city some years ago, he told me of the value of tin and gold as a filling-material, and asked me to try it, but did not go into particulars as to its special advantages or methods of working. At that time, in weak teeth of adults and in children's teeth, I was using tin, either foil or Robinson's metal, to some extent, and in some doubtful cases, instead of these, I tried the tin-gold accordingly to my own ideas as to how it should be used. From time to time I saw these fillings; and their appearance encouraged me to more extensive use, until now I fill several cavities each day with it. I am led to speak of it to-night, not to claim any ideas or methods, but simply to call your attention to it and ask you to give it a trial. I use it where formerly I should have used cement and gutta-percha, and not in cavities where gold will stand, except those in the crown of the upper wisdom teeth.

In all my work with tin-foil I have seen decay come only twice, which meant undoubtedly defective work. I was very clumsy in my earlier efforts, not having had such a knowledge of the working of soft gold as many of you have, and from that reason two or three fillings came out piece by piece; but continued practice soon remedied that, and now I find tin-gold to be as easy and sure to work as gutta-percha. The theory and advantages of this method are admirably set out by Dr. Miller in the May, 1889, number of the *Dental Cosmos*, and his article will tell you far more than I can of my own knowledge.

I use it chiefly in crown cavities, in molars for young persons and the wisdom teeth of adults; in small approximal cavities back of the cuspid for all ages, and in nearly all buccal and palatal cavities. I never put in a gold filling of any size in an approximal cavity in the bicuspids or molars without a few pieces of tin-gold at the cervical wall. I often fill two-thirds of the cavity with tin-gold and then build on a grinding surface with crystal gold or annealed cylinders. I top the tin-gold out with gold in this way for appearance's sake, and I also feel surer of my contour; although with more experience I should have no fear of the latter not being perfect.

I use it with wedge-shaped soft-foil pluggers, and prepare it for each cavity by rolling a part of a sheet of tin inside the same proportional part of a sheet of gold and cutting it off so as to leave the pieces diamond-shaped. There is a foil-trimmer that belongs with the Harvard set of pluggers that I find very useful in condensing.

I will read a few extracts from Dr. Miller's paper, and in conclusion ask you to try tin-gold. I feel that it has made life easier and my work much more satisfactory to myself.—*International Dental Journal.*

---

